# Anti-neoplastic characteristics and potential targets of calycosin against bisphenol A-related osteosarcoma: bioinformatics analysis

**DOI:** 10.1080/21655979.2021.1956401

**Published:** 2021-07-26

**Authors:** Qijin Pan, Ka Wu, Jiachang Tan, Yu Li, Xiao Liang, Min Su

**Affiliations:** a Department of Oncology, Guigang City Peoples’ Hospital, The Eighth Affiliated Hospital of Guangxi Medical University, Guigang, Guangxi, PR China; b Department of Pharmacy, The Second People’s Hospital of Nanning City, the Third Affiliated Hospital of Guangxi Medical University, Nanning, China; c Department of Bone and Soft Tissue Surgery, Affiliated Tumor Hospital, Guangxi Medical University, Nanning, PR China; d Laboratory of Environmental Pollution and Integrative Omics, Guilin Medical University, Guilin, PR China

**Keywords:** Osteosarcoma, Bisphenol A, calycosin, network pharmacology, molecular docking, findings

## Abstract

Environmentally, bisphenol A (BPA) is a well-known pollutant caused human health risk, including osteosarcoma (OS). OS, a deadly bone neoplasia, may occur in children and adults. However, the anti-OS pharmacotherapy prescribes limitedly in clinical practice. Interestingly, previous experimental evidences indicate calycosin-exerting potential anti-OS actions. Thus, in this report, we aimed to further characterize and detail the therapeutic targets and molecular mechanisms of calycosin-anti-BPA-related OS by using network pharmacology and molecular docking analyses. In results, the bioinformatics data disclosed all mapped, core targets, biological functions, molecular pathways of calycosin to treat BPA-related OS. The computational analysis using molecular docking indicated that potential binding ability of core targets in calycosin to treat BPA-related OS was identified. Moreover, detailed biological functions and optimal pathways of calycosin-anti-BPA-related OS were revealed, as shown in integrated network maps. Taken together, these network pharmacology and structural biology findings illustrate the core biotargets, pharmacological functions and pathways of calycosin-anti-BPA-related OS. Potentially, these core targets identified by molecular docking may attribute to the potential clinical application of calycosin against BPA-related OS.

## GRAPHICAL ABSTRACT

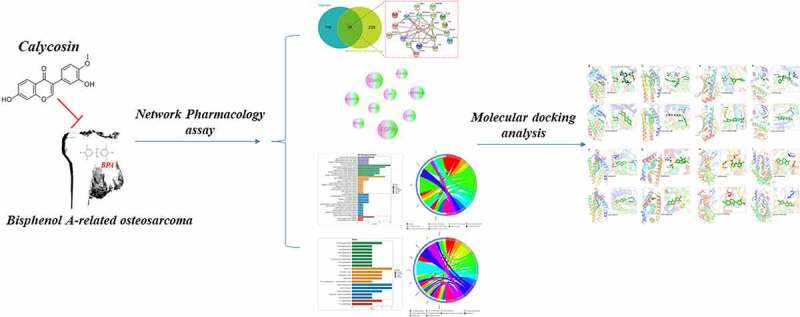

## 1. Background

OS, one kind of malignant bone cancers, may occur in adolescent that this neoplasm affects health and life [1]. Statistically, the incidence of OS is increasing yearly in China because of the huge population and increased newborns [2]. It is reported that the etiology of OS may be associated with hereditary factor, food habit, and environmental exposure [3]. In recent decades, the environmental pollution is of great concerns as ubiquitous pollutants are found with inducing potential human health risks [4]. BPA, a synthetic material used widely, is a well-reported endocrine disrupting chemical that may induce potential reproductive impairment, immunological and neurological dysfunctions, and tumorigenesis [5]. It is defined that BPA may be a carcinogen, such as breast cancer and prostate cancer, as issued by International Agency for Research on Cancer and the National Toxicology Program [6]. Furthermore, BPA exposure is found with potential risk of developing of OS in experimental reports [7,8]. Currently, there is no existing medicine to treat BPA- related OS, especially natural compounds.

Calycosin, a naturally producing ingredient, is reported with functionally exerting effective antioxidation, neuroprotection, anti-cancers [8]. Preclinically, calycosin is pharmacologically evidenced with beneficial actions against malignant cancers, such as colorectal cancer [9], hepatocellular carcinoma [10]. The previous study shows the calycosin-anti-osteosarcoma effect *in vitro*, characterized with pharmacological actions of reducing cell proliferation, and promoting cell apoptosis [11]. However, the bioinformatic and experimental investigations of calycosin against BPA-related OS remain unreported. Interestingly, network pharmacology counterplan is effectively used for detection and identification of hug biotargets and molecular mechanisms of bioactive agent to treat disease [12,13]. Our previous findings using network pharmacology have achieved, including vitamin C against leukemia, niacin against coronavirus disease-2019 [14,15]. To attain current aim, this bioinformatic report using network pharmacology and molecular docking assays was designed to detect and characterize all anti-BPA-related OS targets and mechanisms of calycosin, a promising phytoestrogen.

## 2. Methods

### 2.1 Detection of functional genes of calycosin and BPA-related OS

A series of analytical tools, including Traditional Chinese Medicine Systems Pharmacology Database (TCMSP), SwissTargetPrediction, Bioinformatics Analysis Tool for Molecular mechANism of Traditional Chinese Medicine (BATMAN-TCM), SuperPred were applied for obtaining anti-disease genes of calycosin, and BPA-related OS genes were harvested by using the databases of GeneCard, Online Mendelian Inheritance in Man (OMIM). In further determination, these isolated genes of calycosin and BPA-related OS were re-tested via online bioinformatics tool for drawing Venn diagram before all mapped targets of calycosin in the treatment of BPA-related OS were obtained [16,17].

### 2.2 Identification of core targets of calycosin in the treatment of BPA-related OS

After assays, these mapped genes of calycosin and BPA-related OS were used to generate a protein–protein interaction (PPI) network of calycosin in the treatment of BPA-related OS by using String tool. And the raw data (saved as tsv. files) were determined via Cytoscape tool to identify core targets. All core targets were screened out according to the Degree value by using NetworkAnalyzer for topological analysis [17,18].

### 2.3 Enrichment analysis of molecular functions and pathways of core targets

By using FunRich analysis tool, the core targets of calycosin in the treatment of BPA-related OS were determined via Functional Annotation Bioinformatics Microarray Analysis (DAVID) database for revealing pharmacological processes and pathways of calycosin in the treatment of BPA-related OS. On the basis of the -Log p-value, top biological processes and signaling pathways of calycosin in the treatment of OS were created and demonstrated [19,20].

### 2.4 Construction of networking visualization

By applying Cytoscape software for summarizing current bioinformatics data, the gene ontology (GO)-associated biological process and molecular pathway for calycosin in the treatment of BPA-related OS were identified accordingly. Furthermore, the visualization graph revealing drug-target-gene ontology-biological process-pathway-disease was constructed [21,22].

### 2.5 Molecular docking verification

As reported previously [23,24], all core targets were verified by molecular docking analysis, and chemical structure of calycosin was obtained from PubChem database. The functional protein structure was collected from the Protein Data Bank (PDB) database. By applying ChemBio Office 2010 software, the docked ligand molecule and the original ligand molecule were determined according to the root mean square deviation (RMSD) before the rationality of docking parameter setting was identified. It was generally referenced that RMSD≤4 Å was analytical threshold for conformation of the functional ligand to match the original ligand after molecular docking determination.

## 3 Results

### 3.1 Preliminary bioinformatics data of targets in calycosin and BPA-related OS

**Figure 1.**
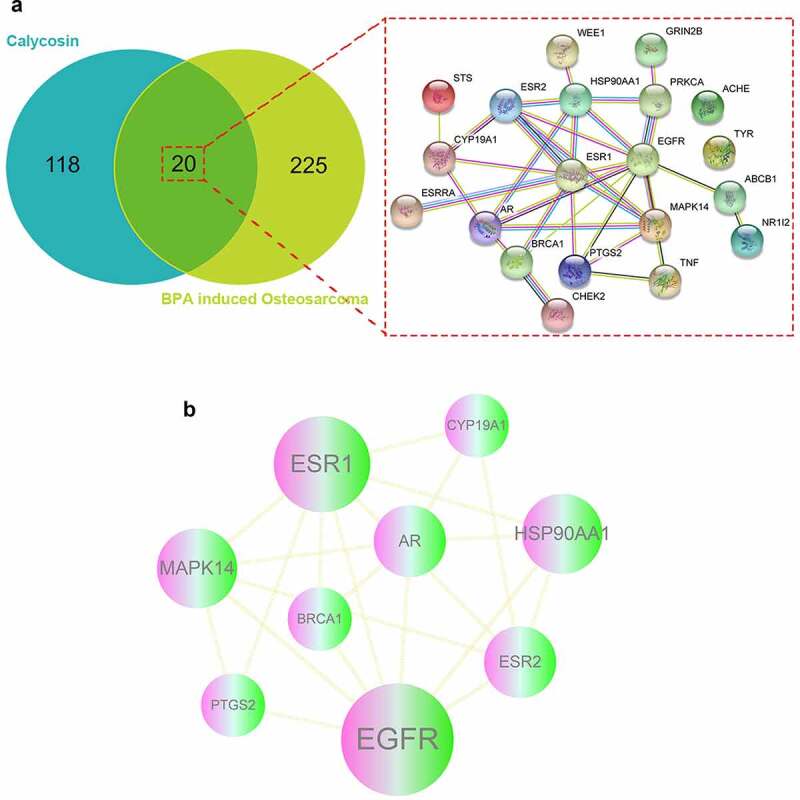
(a) As showed in Venn diagram assay, all candidate, mapped targets of calycosin and BPA-related OS were identified and produce network map using shared targets. (b) After further bioinformatics analysis, all 9 core targets of calycosin in the treatment of BPA-related OS were identified accordingly

As results, a number of 245 BPA-related OS genes were acquired, and other 138 anti-disease genes of calycosin were attained accordingly. As shown in Venn graph, a total of 20 mutual genes of calycosin and BPA-related OS were identified and highlighted in interaction network for connected visualization (Figure 1a).

### 3.2 Findings of all core targets in calycosin in the treatment of BPA-related OS

The mutual genes were further assayed by using Cytoscape software. The topological data showed that median degree of freedom was 3.667, and maximum degree of freedom was 10. Therefore, the core target screening range was set as 4–10. As a result, 9 core targets of calycosin in the treatment of BPA-related OS were identified totally, including Epidermal growth factor receptor erbB1 (EGFR), Estrogen receptor alpha (ESR1), Heat shock protein HSP 90-alpha (HSP90AA1), Mitogen-activated protein kinase 14 (MAPK14), Estrogen receptor beta (ESR2), Androgen receptor (AR), Breast cancer type 1 susceptibility protein (BRCA1), Prostaglandin G/H synthase 2 (PTGS2), Cytochrome P450 19A1 (CYP19A1). The findings were illustrated in an interaction network (Figure 1b) and more information was detailed in Supplemental Table 1.

### 3.3 Enrichment analysis findings of core targets

**Figure 2.**
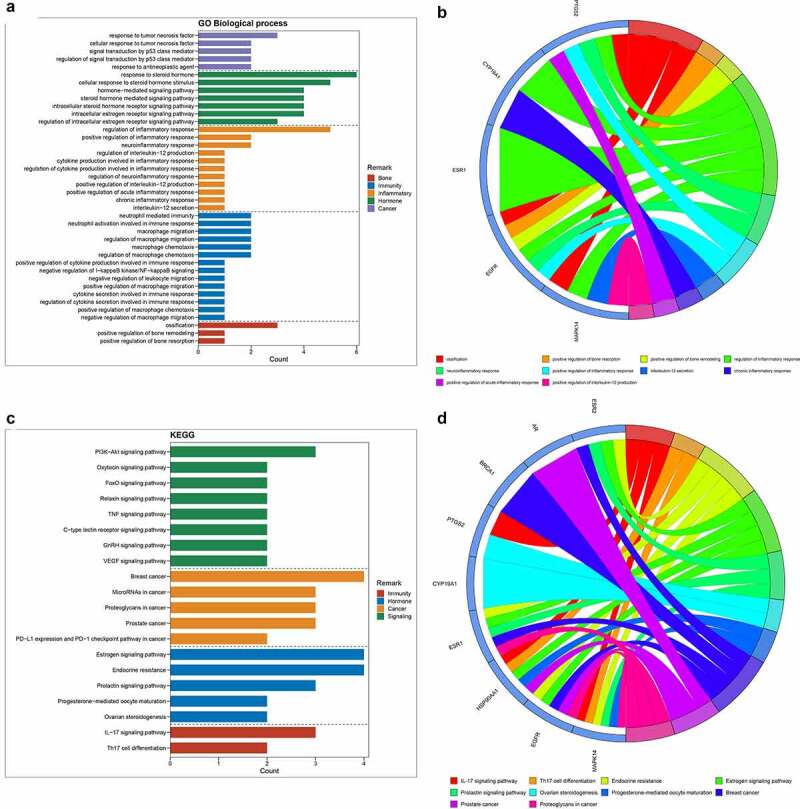
(a) After enrichment analyses, top GO-based functional processes, including bone, immunity, inflammation, hormone and cancer, were revealed. (b) Other top KEGG-based molecular mechanisms of calycosin to treat BPA-related OS, including immunity, hormone, cancer and signaling, were uncovered

All 9 core targets/genes were used to conduct GO-associated function and Kyoto Encyclopedia of Genes and Genomes (KEGG) pathway enrichment analyses through *R* language-related packages. As results, the histogram and Circos circle chart revealing GO-based biological processes were presented in Figure 2a,b; and then the histogram and Circos circle chart uncovering KEGG signaling pathways were showed in Figure 2c,d. The enrichment results suggested that the biological processes (BP) were mainly involved in ossification, positive regulation of bone resorption, positive regulation of bone remodeling, regulation of inflammatory response, neuroinflammatory response, positive regulation of inflammatory response, interleukin-12 secretion, chronic inflammatory response, positive regulation of acute inflammatory response, positive regulation of interleukin-12 production, regulation of neuroinflammatory response, regulation of cytokine production involved in inflammatory response, cytokine production involved in inflammatory response, regulation of interleukin-12 production, regulation of macrophage chemotaxis, macrophage chemotaxis, regulation of macrophage migration, macrophage migration, negative regulation of macrophage migration, positive regulation of macrophage chemotaxis, regulation of cytokine secretion involved in immune response, cytokine secretion involved in immune response, positive regulation of macrophage migration, neutrophil activation involved in immune response, neutrophil mediated immunity, negative regulation of leukocyte migration, negative regulation of I-kappaB kinase/NF-kappaB signaling, positive regulation of cytokine production involved in immune response, response to tumor necrosis factor, response to antineoplastic agent, regulation of signal transduction by p53 class mediator, signal transduction by p53 class mediator, cellular response to tumor necrosis factor, response to steroid hormone, intracellular estrogen receptor signaling pathway, cellular response to steroid hormone stimulus, intracellular steroid hormone receptor signaling pathway, regulation of intracellular estrogen receptor signaling pathway, steroid hormone mediated signaling pathway, hormone-mediated signaling pathway (Supplemental Table 2). The other 26 KEGG molecular pathways of all core targets (*P* < 0.05) were identified with involvement of IL-17 signaling pathway, Th17 cell differentiation, Endocrine resistance, Estrogen signaling pathway, Prolactin signaling pathway, Ovarian steroidogenesis, Progesterone-mediated oocyte maturation, Breast cancer, Prostate cancer, Proteoglycans in cancer, MicroRNAs in cancer, PD-L1 expression and PD-1 checkpoint pathway in cancer, VEGF signaling pathway, GnRH signaling pathway, C-type lectin receptor signaling pathway, PI3K-Akt signaling pathway, TNF signaling pathway, Relaxin signaling pathway, FoxO signaling pathway, Oxytocin signaling pathway (Supplemental Table 3).

### 3.4 Interaction network findings

**Figure 3.**
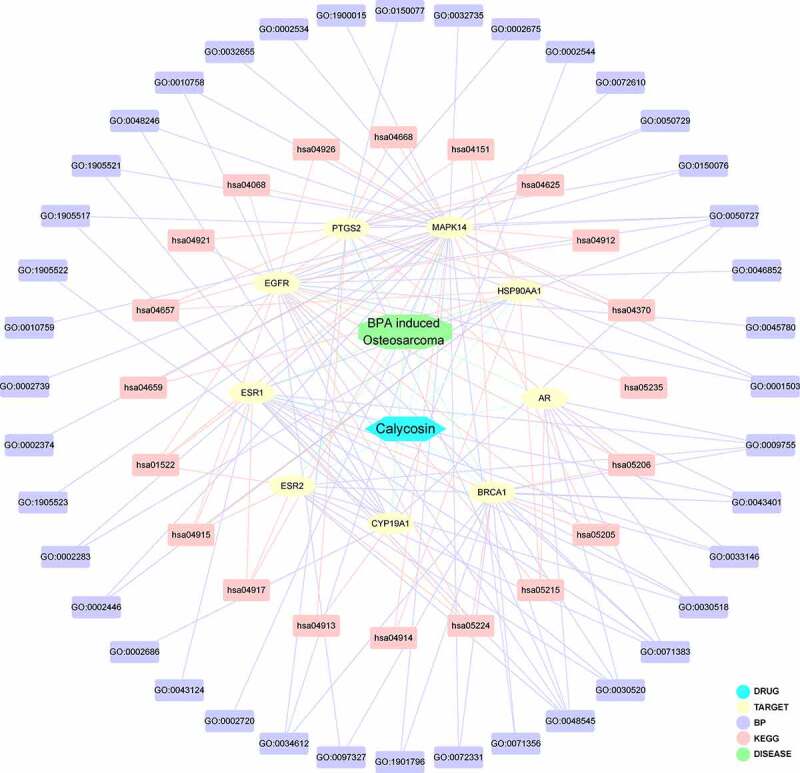
By using integrated analysis, network visualization of calycosin-target-BP-KEGG-BPA/OS was produced and highlighted

**Figure 4.**
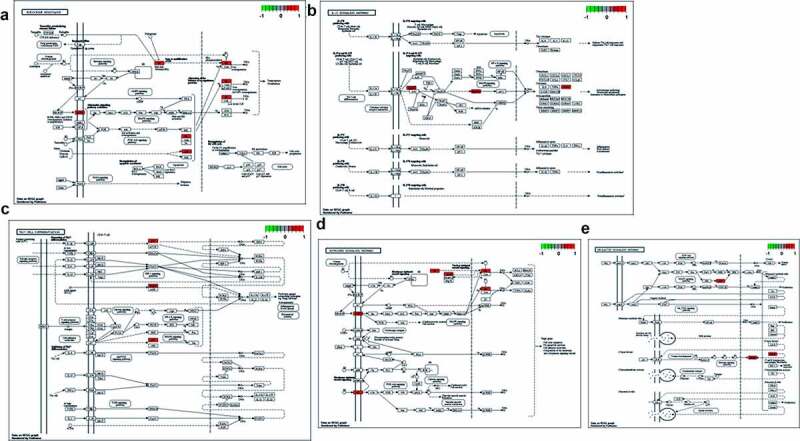
The core targets enriched in KEGG signaling pathway were identified by suing R-language software, as shown in red marks

The network visualization of calycosin-target-BP-KEGG-BPA-related OS was determined and highlighted through Cytoscape software, as revealed in Figure 3. The core targets enriched in KEGG signaling pathway were identified in Figure 4 by using R-language software, as highlighted in red marks.

### 3.5 Molecular docking findings

**Figure 5.**
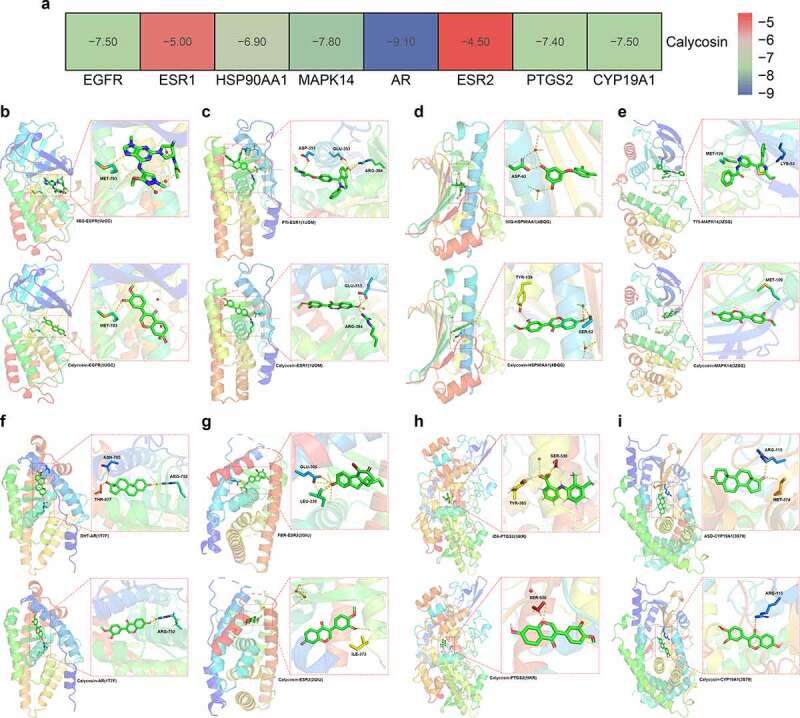
(a) The binding energy data in core genes were showed in details. (b) Molecular docking findings revealed the biological structure features of core proteins/targets in details

By using PDB-based analysis, BRCA1 protein/target was excluded as this protein failed to dock with calycosin-associated ligands. As shown in binding energy data (Figure 5a), EGFR (PDB ID: 5UGC) with RMSD of original ligand 8BS was 3.889 Å, and its hydrogen bond with 5UGC protein acted on amino acid residue MET-793 (2.9 Å). And hydrogen bond between calycosin and amino acid residue MET-793 (3.0 Å) was formed effectively (Figure 5b). In ESR1 (PDB ID:1UOM), the RMSD of original ligand PTI was 3.645 Å, in which hydrogen bonded with 1UOM protein to amino acid residues ASP-351 (3.5 Å), GLU-353 (2.7 Å), ARG-394 (3.0 Å). And calycosin formed a hydrogen bond with amino acid residues GLU-353 (1.8 Å) (Figure 5c). In HSP90AA1 (PDB ID: 4BQG), the RMSD of original ligand 50Q was 3.105 Å, and the hydrogen bonded with 4BQG protein to amino acid residue ASP-93 (2.7 Å). And Calycosin and the amino acid residue SER-52 (3.5 Å)), TYR-139 (3.1 Å) formed hydrogen bond (Figure 5d). In MAPK14 (PDB ID: 3ZSG), the RMSD of original ligand T75 was 3.789 Å, and its hydrogen bonded with the 3ZSG protein acted on amino acid residues LYS-53 (3.2 Å), MET-109 (2.7 Å). And calycosin and amino acids residue MET-109 (3.4 Å) formed hydrogen bond (Figure 5e). In AR (PDB ID: 1T7F), the RMSD of original ligand DHT was 0.0004796 Å, in which hydrogen bonded with the 1T7F protein to the amino acid residues ASN-705 (2.7 Å), ARG-752 (3.0 Å), THR-877 (2.8 Å). And calycosin formed a hydrogen bond with amino acid residue ARG-752 (2.1 Å) (Figure 5f). In ESR2 (PDB ID: 2GIU), the RMSD of original ligand FBR was 2.322 Å, in which hydrogen bonded with 2GIU protein to amino acid residues GLU-305 (2.5 Å), LEU-339 (3.6 Å). And calycosin formed hydrogen bond with amino acid residue ILE-373 (2.6 Å) (Figure 5g). In PTGS2 (PDB ID: 5IKR), the RMSD of original ligand ID8 was 2.874 Å, and the hydrogen bond with 5IKR protein acted on amino acid residues TYR-385 (2.0 Å), SER-530 (2.0 Å). And calycosin formed hydrogen bond with amino acid residue SER-530 (2.9 Å) (Figure 5h). In CYP19A1 (PDB ID: 3S79), the RMSD of original ligand ASD was 0.0004888 Å, in which hydrogen bonded with 3S79 protein to amino acid residues ARG-115 (3.3 Å), MET-374 (2.8 Å). And calycosin formed hydrogen bond with amino acid residue ARG-115 (3.1 Å) (Figure 5i).

## 4 Discussion

In current bioinformatics study, we identified and elucidated the pharmacological activity and mechanism of calycosin in the treatment of BPA-related OS. More interestingly, all core targets of calycosin in the treatment of BPA-related OS were revealed, comprising EGFR, ESR1, HSP90AA1, MAPK14, ESR2, AR, BRCA1, PTGS2, CYP19A1. In details, the biological functions enriched with core targets were identified with regulatory processes of bone, immunity, inflammation, hormone and cancer for revealing calycosin-exerted BPA-related OS activities. EGFR, if mutated, is reportedly correlated with the pathological development of tumorigenesis [25]. Another reporting evidence shows that BPA induces cancer cell proliferation via activation of EGFR activity [26]. ESR1, also known as estrogen receptor alpha, is medically detected with mutation in many human cancer samples, such as breast cancer [27]. It is reported that high BPA exposure in urine samples may be related to human non-small cell lung cancer via modifying ESR1 genetic polymorphism [28]. HSP90AA1 can act on the functions in invasion and migration of cancer cells, and it is closely related to poor prognosis of tumors [29]. HSP90AA1, functioning as a key promotor of autophagy, is positively associated with the development of osteosarcoma chemoresistance [30]. MAPK14 may act as an integration protein for multiple biochemical events, and it is involved in multifarious cellular processes including cell differentiation and proliferation, gene transcription regulation [31]. Increasing evidences suggest that MAPK14 activity may activate the cancer cell migration, invasiveness and angiogenesis [32]. AR, a functional androgen receptor, is a therapeutic target used in cancers, such as prostate tumor. And anti-cancer action is achieved through regulating androgen receptor signaling [33]. It is reported that BPA exposure regulates specific gene expressions, such as estrogen receptor beta, in human prostate cancer cells for androgen-dependent proliferation [34]. BRCA1, a nuclear phosphoprotein, exerts a physiological action in maintaining genomic stability, and it also functions as a cancer suppressor [35]. It is found that BRCA1 may suppress the BPA-induced human breast cancer cell proliferation *in vitro* and *in vivo* [36]. The transcriptional data of prostaglandin endoperoxide synthase (PTGS) signaling characterizes the key roles on biological event of the tumor-related microenvironment, such as inflammatory infiltration [37]. The clinical trial analysis suggests the potential impact of CYP19A1 in postmenopausal endocrine responsive breast cancer in humans [38]. The study *in vitro* indicates that BPA induces cell proliferation and growth in human choriocarcinoma cell line through affecting estradiol metabolism [39]. As revealed in computational analysis, the pharmacological actions of calycosin in the treatment of BPA-related OS were identified in details, such as ossification, positive regulation of bone resorption, positive regulation of bone remodeling, regulation of inflammatory response, neuroinflammatory response, positive regulation of inflammatory response, interleukin-12 secretion, positive regulation of interleukin-12 production. These bioinformatics findings indicate that calycosin may play potential anti-BPA-related OS activities by functionally modulating enrichment-assayed molecular processes. In further investigation, the KEGG enrichment analysis-based findings revealed all calycosin-anti-BPA-related OS mechanisms, including IL-17 signaling pathway, Th17 cell differentiation, Endocrine resistance, Estrogen signaling pathway, Proteoglycans in cancer, MicroRNAs in cancer, PD-L1 expression and PD-1 checkpoint pathway in cancer, VEGF signaling pathway, GnRH signaling pathway, PI3K-Akt signaling pathway, TNF signaling pathway, FoxO signaling pathway. The current pathway enrichment analysis indicated that calycosin-exerted anti- BPA-related OS pharmacological mechanisms were achieved in immunity, hormone, cancer and signaling regulations. More notably, these computational evidences of GO-based molecular processes were consistent with bioinformatics findings of KEGG signaling pathways of calycosin against BPA-related OS. In current limitations, *in-silico* findings using molecular docking analysis should be validated in clinical and experimental studies before the future clinical application of calycosin to treat BPA-related OS will be achieved.

## 5 Conclusion

In conclusion, our current computational findings identify detailed pharmacological biotargets, biological processes, molecular mechanisms of calycosin to treat BPA-related OS. In future medical application, naturally-isolating calycosin will be applied in clinical practice against osteosarcoma, including BPA-related OS.

## Supplementary Material

Supplemental Material

## Notes

1. Mapped, core targets, biological functions, molecular pathways of calycosin to treat BPA-related OS were revealed.
2. Computational analysis indicated molecular docking ability and characteristic of core targets in calycosin to treat BPA-related OS
3. Top biological functions of calycosin-anti-BPA-related OS were revealed in details.
4. Top signaling pathways of calycosin-anti-BPA-related OS were uncovered in details.
5. Calycosin may be a promising compound for treating BPA-related OS.
